# From Waste to Worth: A Multi-Study Investigation of Chinese Consumers’ Purchase Intentions Toward Near-Expired Bread

**DOI:** 10.3390/foods15081369

**Published:** 2026-04-15

**Authors:** Ran Gao, Haixiu Gao, Zhaokang Liu, Guangyan Cheng

**Affiliations:** Institute of Food and Nutrition Development, Ministry of Agriculture and Rural Affairs, Beijing 100081, China; 82101231395@caas.cn (R.G.); gaohaixiu@caas.cn (H.G.); 821012550772@caas.cn (Z.L.)

**Keywords:** near-expired food, food waste avoidance, green consumption, information framing, purchase intentions

## Abstract

Reducing food waste and promoting green consumption have emerged as critical priorities in the transition toward a more sustainable food system. Purchasing near-expired food (NEF) offers a pathway to address both issues simultaneously, yet the mechanisms underlying consumers’ intentions toward such products remain underexplored. This research investigates these mechanisms through two complementary studies conducted in China, focusing on near-expired bread as a representative product category. Study 1 (*N* = 1154) draws on the stimulus–organism–response (SOR) framework to examine how key factors shape consumers’ purchase intentions toward near-expired bread. The results show that price discounts and longer remaining shelf life increase purchase intentions by enhancing perceived value and reducing perceived risk. Moreover, consumers’ normative beliefs with regard to food waste avoidance positively predict purchase intentions through heightened moral satisfaction. Study 2 (*N* = 746) employs a 2 × 3 between-subjects factorial experiment to test two types of retail interventions for near-expired bread: discount messages (50% vs. 10% off) and information framing (gain-framed vs. loss-framed). Extending Study 1, this experiment introduces two additional dependent variables—product attitudes and perceived environmental external benefits—to capture a broader range of consumer responses. ANCOVA results reveal that consumers with higher environmental concern exhibit stronger purchase intentions, more favorable product attitudes, and greater perceived environmental external benefits. Price discount messages significantly influence purchase intentions and product attitudes, whereas information framing affects purchase intentions and environmental external benefits. Notably, the two interventions interact to shape consumers’ perceptions of environmental external benefits. Together, these studies advance a comprehensive understanding of near-expired bread purchases and offer empirical guidance for designing effective retail communication strategies to promote green consumption and reduce food waste.

## 1. Introduction

Globally, one-third of the food produced annually (1.3 billion tons) is wasted [1]. This wastage not only depletes natural resources and contributes to greenhouse gas emissions, but also represents significant economic losses along the supply chain. In the entire food waste chain, the consumption stage accounts for the largest proportion of food waste, representing approximately 35% of the total [2]. A significant driver of this waste at the consumer level is the rejection of so-called “suboptimal food”. Suboptimal food refers to food products that deviate from normal food in terms of date labeling (i.e., NEF), cosmetic specifications (i.e., shape), or packaging (i.e., damaged packaging), while retaining their intrinsic quality and safety [3]. This study focuses on one type of NEF, namely near-expired bread with a shelf life of within one week. Bread was selected because China’s bread market has expanded substantially in recent years, driven by shifting dietary habits, food industry modernization, and urbanization. According to industry data, the country’s bread baking market reached approximately 116 billion yuan in 2025, supported by over 330,000 bakery stores nationwide [4]. Given bread’s short shelf life and high turnover frequency [5], this market expansion has made near-expired bread a commonly encountered suboptimal product for Chinese consumers. This prevalence is reflected in consumer behavior: among near-expired food categories accepted and purchased by Chinese internet users in 2021, bread and pastries ranked second (33.6%), trailing only packaged snacks [6].

China’s NEF market has developed rapidly [7] and its fast growth has occurred within a regulatory context that remains underdeveloped, fueling growing consumer concerns over quality and safety—even though these products retain their intrinsic quality and safety. Meanwhile, the NEF market is also associated with substantial food waste. Consumer purchasing behavior lies at the heart of this dilemma: their willingness to buy NEF directly determines whether these products are sold or discarded, while their safety concerns shape that willingness. Understanding the factors that influence this purchasing decision is therefore critical—not only for mitigating food waste and promoting green consumption, but also for guiding policy and marketing strategies in this emerging market.

Despite growing scholarly attention to near-expired food (NEF) [8,9,10,11,12], the literature remains fragmented. Existing studies have examined price discounts, remaining shelf life, and normative beliefs in isolation, leaving the interplay among these factors largely unexplored. Moreover, the effects of price discounts remain inconclusive—particularly regarding different discount levels and their interaction with information framing—and empirical evidence from the Chinese market, where the NEF sector is expanding rapidly, remains scarce. To address these gaps, this study applies the stimulus–organism–response (SOR) framework to integrate fragmented factors, investigates the interactive effects of discount levels and information framing through a factorial experiment, and focuses on near-expired bread in the Chinese context.

The remainder of this paper is organized as follows. After a review of the literature and the development of research hypotheses, we present the methods and results of two empirical studies, followed by a discussion and practical implications.

## 2. Literature Review

### 2.1. Factors Influencing Consumers’ Near-Expired Food Purchase Intention

Previous studies have identified multiple factors that influence consumers’ purchase intentions toward NEF. These can be categorized into product-related factors, consumer-related factors, and situational factors.

#### 2.1.1. Product-Related Factors: Price Discounts and Remaining Shelf-Life

Price discounts have been extensively examined as a key determinant of NEF purchase intentions. From a theoretical perspective, price serves as a dual signal in consumer decision-making: it not only represents economic sacrifice but also conveys information about product quality [13]. This dual role becomes particularly salient in the context of NEF, where consumers must weigh the benefit of lower prices against potential quality concerns.

On the one hand, price discounts can mitigate consumers’ reluctance to purchase NEF. Consumers tend to prioritize non-NEF alternatives in routine shopping decisions [8], largely due to concerns about the reduced value of short-dated food [9]. Discounts help offset these concerns by providing economic compensation for perceived value decline [9]. On the other hand, price reductions may backfire. When consumers encounter discounted NEF, they may infer that the price cut signals deteriorating quality [14], potentially generating negative impressions and posing reputational risks to retailers [15]. This inference is consistent with the price-quality heuristic documented in consumer behavior literature [13]. These mixed findings suggest that the effect of price discounts on NEF purchase intention is not straightforward—it depends on how consumers interpret the discount. While some consumers view it as a fair trade-off for shorter remaining shelf life, others perceive it as a warning sign of compromised quality. This interpretive ambiguity points to the need for a more nuanced understanding of the psychological mechanisms underlying consumers’ responses to discounted NEF. Moreover, beyond the discount itself, contextual factors such as the timing of price adjustments may further complicate consumer reactions [16], an aspect that remains underexplored in the current literature.

The second product-related factor is remaining shelf life. Consumers perceive higher risks associated with NEF compared to non-NEF, and this risk perception intensifies as the number of remaining shelf-life days decreases [10,17]. Notably, more experienced consumers tend to check shelf life more frequently [17], suggesting that product knowledge may moderate the relationship between remaining shelf-life and purchase intentions. However, the psychological mechanisms underlying this shelf-life effect—whether it operates through perceived quality, perceived health risk, or anticipated regret—remain underexplored.

#### 2.1.2. Consumer-Related Factors: Ethical Concerns and Social Image

Beyond product attributes, consumer characteristics also shape NEF acceptance. One stream of research highlights the role of ethical concerns. Consumers with higher environmental awareness are more willing to accept NEF, viewing it as a means to reduce food waste [11]. This ethical motivation can be strengthened through interventions that enhance consumers’ understanding of shelf-life labels and raise their awareness of environmental issues [18]. Retailers have also leveraged this by combining discount strategies with messaging about environmental benefits [19]. These findings suggest that NEF purchase decisions involve both economic calculation and moral consideration. More broadly, researchers have conceptualized this as a tension between two value orientations: egoism, which drives consumers to seek discounted prices, and altruism, which motivates them to avoid food waste.

Another consumer-related factor is social image concern. Some consumers avoid purchasing NEF due to fears of being perceived as overly stingy, as buying such products may carry social stigma [20]. This reluctance to be deemed frugal leads consumers to reject NEF to maintain their social reputation, even when they have no personal quality concerns. This social dimension of NEF consumption remains under-researched compared to the economic and ethical dimensions.

#### 2.1.3. Situational Factors

In addition to product and consumer factors, situational variables may moderate NEF purchase decisions. For instance, Chung et al. [21] found that consumers’ sensitivity to NEF varies by time of day, with lower sensitivity in the evening than in the morning. This finding points to the role of contextual factors—such as time pressure, shopping goals, or mood—in shaping NEF acceptance. However, research on situational moderators remains fragmented, and theoretical integration is lacking.

### 2.2. Theoretical Framework: The Stimulus–Organism–Response (SOR) Model

SOR has emerged as a foundational theoretical lens for understanding how environmental cues shape individual behavior. Originally proposed by Woodworth in 1926, the framework conceptualized that external stimuli could elicit individual responses, with personal factors serving as mediating or moderating variables in this process [22]. Building on this foundation, Belk [23] refined the framework in 1975 by formally identifying “situation” as a distinct construct that precedes behavior, thereby establishing the tripartite structure of stimulus–organism–response that remains influential today. In summary, the S-O-R framework posits that external stimuli (S) trigger individuals’ internal states (O), which in turn shape their behavioral responses (R) [22,23]. Within the domain of food consumption and sustainable behavior, the SOR framework has gained increasing traction. Studies have applied the framework to examine consumers’ responses to organic food labels [24], sustainable packaging [25], and food waste reduction interventions [26]. In the context of near-expired food (NEF) consumption, the SOR framework offers a coherent theoretical lens to integrate the fragmented factors identified in prior literature.

### 2.3. Post-Purchase Outcomes: Waste Prevention Behaviors

Beyond the purchase decision itself, scholars have also examined the consequences of NEF consumption, particularly its impact on household food waste. On one hand, consumers’ purchase of NEF represents an ethical and practical practice for reducing food waste and advancing sustainability [12]. Zhang et al. [27] found that after purchasing NEF, consumers adopt more waste-prevention behaviors for these products than for other food categories, regardless of whether they receive information on food waste avoidance. This suggests that the act of purchasing NEF may itself activate heightened awareness of waste. On the other hand, the relationship between NEF consumption and waste reduction is not straightforward. Retailers’ discounting and price-cutting practices may inadvertently trigger consumer overconsumption, which in turn generates food waste at the household level [28]. This paradox highlights a critical tension: while discounts encourage the purchase of NEF (thereby reducing retail waste), they may also lead to excess purchasing and subsequent household waste. This duality calls for a more holistic understanding of NEF’s net impact on the food system.

### 2.4. External Interventions: Information and Labeling Strategies

Given the complexity of consumer responses to NEF, researchers have explored interventions that might facilitate more favorable attitudes and behaviors. One promising approach involves information labeling. Szymkowiak et al. [29] conducted an experiment in which NEF was labeled as “zero-waste,” comparing consumers’ attitudes toward labeled NEF, unlabeled NEF, and non-NEF. Their findings suggest that such labeling can positively influence consumer perceptions. Similarly, Zhang et al. [27] found that adding food waste avoidance labels to NEF enhances consumers’ moral satisfaction, which in turn reduces household waste of such food. This line of research points to the potential of labeling strategies to address both the pre-purchase hesitation and post-purchase waste challenges associated with NEF. However, the mechanisms through which such labels operate—whether through moral elevation, guilt reduction, or increased salience of environmental consequences—remain underexplored.

Overall, the literature has identified a range of factors influencing consumers’ NEF purchase intentions—including product attributes (price, remaining shelf life), consumer characteristics (ethical concerns, social image), and situational variables—while also beginning to examine post-purchase outcomes and informational interventions. Despite these advances, three gaps remain. First, these factors have largely been examined in isolation, resulting in a fragmented understanding of how they jointly shape purchase intentions. Second, while price discounts are widely recognized as a key determinant, empirical findings remain mixed; the differential effects of varying discount levels and their interaction with information framing remain underexplored. Third, most NEF research has been conducted in developed economies, leaving the rapidly expanding Chinese market largely understudied. This study is designed to address these limitations in three ways. First, it advances a coherent theoretical framework by integrating fragmented factors within the SOR paradigm, moving beyond isolated examinations of individual variables. Second, it employs a 2 × 3 factorial experiment to systematically examine how discount levels interact with information framing—a question that prior mixed findings have left unresolved. Third, it contributes empirical evidence from the Chinese context, a rapidly growing NEF market that has received limited attention in the existing literature. Together, these design features enable a more comprehensive understanding of consumer responses to near-expired bread.

## 3. Theoretical Background

### 3.1. Predictors from S-O-R Framework

**Figure 1:**
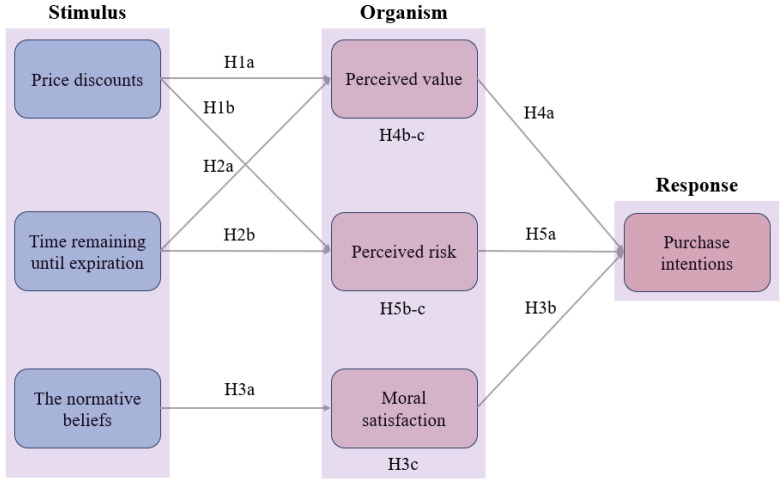
Theoretical framework.

Study 1 adopts the stimulus–organism–response (SOR) framework as its overarching theoretical lens. In the context of NEF consumption, we conceptualize price discounts, time remaining until expiration, and normative beliefs with regard to food waste avoidance as stimulus variables. These external cues influence consumers’ internal evaluations—specifically perceived value, perceived risk, and moral satisfaction—which subsequently determine their purchase intentions toward NEF. The final hypothesized model of factors influencing consumers’ intentions of purchasing near-expired bread is illustrated in Figure 1.

#### 3.1.1. Price Discounts

Price discounts are a primary marketing strategy for NEF, designed to incentivize consumer purchases [30]. Within the SOR framework, discounts serve as an external stimulus that activates consumers’ cognitive and affective evaluations. Drawing on perceived value theory, price discounts enhance consumers’ perception of economic benefits, thereby increasing the perceived value of NEF [30,31]. This enhanced perceived value, in turn, positively influences purchase intentions. Simultaneously, price discounts may reduce consumers’ perceived risk associated with NEF. Given consumers’ tendency to harbor quality concerns about near-expired products [32], discounts can serve as a risk-mitigating cue, lowering psychological barriers to purchase [33]. Thus, price discounts operate through both benefit-enhancing and risk-reducing pathways.

However, the magnitude of the discount may matter. A nominal discount may be insufficient to offset quality concerns, while a substantial discount may effectively compensate for perceived value loss [33]. Based on on-site observations in Chinese retail settings, this study operationalizes high and low discount levels as 50% and 10% off the original price, respectively. Accordingly, we propose:

**H1a.**  *Price discounts positively influence consumers’ perceived value of near-expired bread*.

**H1b.**  *Price discounts negatively influence consumers’ perceived risk of near-expired bread*.

**H1c.**  *Price discount messages for near-expired bread will have a significant positive impact on consumers’ purchase intentions*.

#### 3.1.2. Time Remaining Until Expiration

Time remaining until expiration serves as another critical stimulus in the SOR framework, shaping consumers’ internal evaluations of NEF. As food approaches its expiration date, consumers rely on this temporal cue to infer product quality and safety [34,35]. Prior research has consistently demonstrated that shorter remaining shelf-life reduces consumers’ perceived value and perceived quality, thereby diminishing purchase intentions [36,37].

The underlying mechanism operates through two interrelated pathways. First, consumers perceive near-expired products as offering diminished utility relative to the time and monetary costs incurred, leading to lower perceived value [38]. Second, despite the objective safety of NEF, consumers associate shorter remaining shelf-life with heightened health risks, resulting in elevated perceived risk [32]. These risk perceptions intensify as the expiration date draws nearer.

Within the SOR framework, time remaining until expiration functions as an external stimulus that negatively influences perceived value and positively influences perceived risk—both of which are organismic states that ultimately drive purchase intentions. Accordingly, we propose:

**H2a.**  *A longer time remaining until expiration of near-expired bread positively influences consumers’ perceived value*.

**H2b.**  *A longer time remaining until expiration of near-expired bread negatively influences consumers’ perceived risk*.

#### 3.1.3. Normative Beliefs with Regard to Food Waste Avoidance and Moral Satisfaction

While consumers may purchase NEF due to price discounts, avoiding food waste constitutes another key motivation. Normative beliefs with regard to food waste serve as another critical stimulus in the SOR framework, referring to individuals’ internal perceptions and recognition of the need to reduce food waste. Consumers with normative beliefs with regard to food waste avoidance tend to pay greater attention to food waste issues and are more likely to purchase NEF in retail settings [33]. Moral satisfaction functions as another critical organism that positively influences consumers’ purchase intentions. Moral satisfaction arises from engaging in behaviors aligned with moral norms, thereby yielding a sense of satisfaction. Purchasing morally congruent food products—such as organic food—can enhance consumers’ moral satisfaction and even amplify their sensory experience of the food itself [39]. This is because individuals directly derive moral satisfaction from performing prosocial acts [40]. Research by Tezer et al. [41] noted that when consumers purchase green food, their positive self-perceptions are similarly strengthened, as such purchases represent socially beneficial behaviors. Thus, when consumers with stronger normative beliefs with regard to food waste purchase NEF, they are more inclined to attain moral satisfaction. When individuals perceive that purchasing NEF contributes to societal and environmental well-being, the psychological concept of self-enhancement motivates them to sustain and further enhance their self-esteem. Gaetner et al. [42] conducted a study involving Chinese and American students, finding that both groups were more eager for self-enhancing feedback compared to other types of feedback. Consequently, individuals tend to pursue outcomes and feedback that align with positive self-perceptions. Thus, the moral satisfaction derived from purchasing NEF further strengthens consumers’ positive self-perceptions, making them more willing to purchase such products. Thus, moral satisfaction positively affects consumers’ purchase intentions on NEF. Accordingly, we propose:

**H3a.**  *Normative beliefs with regard to food waste avoidance will positively influence consumers’ moral satisfaction*.

**H3b.**  *Moral satisfaction will positively influence consumers’ purchase intentions*.

**H3c.**  *The normative beliefs with regard to food waste avoidance will increase consumers’ purchase intentions through the increased moral satisfaction*.

#### 3.1.4. Perceived Value

Perceived value serves as another critical organism in the SOR framework. Perceived product value largely impacts consumers’ purchase intentions. Sirdeshmukh et al. [43] argued that perceived value is a subjective and individualized concept, representing the perception of the overall value of a specific product or service. When it comes to food products, quality evaluation dimensions encompass both intrinsic and extrinsic attributes—specifically, the trade-off between a product’s inherent value and its external price [44].

Kim et al. [45] argued that perceived value is the decision reached by consumers after weighing perceived benefits against perceived sacrifices. It also reflects a comprehensive consideration of benefit value, costs, and risks. However, compared to non-NEF items, consumers’ perceived value of near-expired products is more susceptible to cost and risk factors. Consumers may perceive discounted NEF as being of inferior taste [46]. Appearance defects of NEF can diminish consumers’ perceived value of such products. For instance, bananas may develop slight brown spots as their expiration date approaches [47]. When consumers perceive a product’s value to be high, it indicates that the benefits derived from the product outweigh its associated costs and risks. Perceived value helps explain consumers’ purchase intentions and behaviors [48]. Accordingly, we propose:

**H4a.**  *Consumers’ perceived value of near-expired bread will directly affect their purchase intentions*.

**H4b.**  *Price discounts will indirectly increase consumers’ purchase intentions through the increased perceived value*.

**H4c.**  *A Longer time remaining until expiration will indirectly increase consumers’ purchase intentions through the increased perceived value*.

#### 3.1.5. Perceived Risk

Risk is a very important concept in economics that represents uncertainty. Bauer [49] first introduced the concept of perceived risk, which has since been widely applied in the fields of marketing and economics. Perceived risk serves as another critical organism in the SOR framework. Bauer posited that perceived risk emerges when the outcome of a consumer’s behavior deviates from initial expectations and yields undesirable consequences. Subsequently, perceived risk was adopted as a core research variable and widely applied in subsequent consumer behavior studies [50,51,52].

As relevant research advanced, subsequent scholars further enriched the connotation of perceived risk based on their empirical findings.

Stone and Grønhaug [53] argued that when examining consumer behavior, greater attention should be devoted to potential negative outcomes that may ensue. Yeung and Morris [54] developed a framework of perceived risk dimensions, incorporating six distinct types of potential losses as follows: physical loss (negative health impacts on consumers), performance loss (adverse impacts of harmful substances in food on its taste and nutritional value), financial loss (costs of replacing spoiled food and expenses for medical treatment due to illness), time loss (time costs associated with food purchases and illness treatment), social loss (social embarrassment caused by serving poor-quality or contaminated food), and psychological loss (consumers’ anxieties and concerns stemming from food safety risks).

Food safety concerns constitute another key barrier to consumers’ purchase of NEF. Consumers are frequently exposed to food safety incidents [55], and they often experience and express excessive anxiety regarding such issues [56]. When it comes to NEF, consumers exhibit heightened attention to and concern over food safety. Consumers not only perceive NEF as being of inferior quality, but may also fear that consuming such products could pose safety risks [57]. Therefore, this research incorporates the construct of perceived risk, which encompasses the quality and health risks that consumers are most concerned about with regard to NEF. Perceived risk not only influences consumers’ food choices but also shapes their daily consumption decisions [55]. Accordingly, we propose:

**H5a.**  *Consumers’ perceived risk of near-expired bread will directly affect their purchase intentions*.

**H5b.**  *Price discounts will indirectly increase consumers’ purchase intentions through the decreased perceived risk*.

**H5c.**  *A Longer time remaining until expiration will indirectly increase consumers’ purchase intentions through the decreased perceived risk*.

### 3.2. Information Intervention Experiments Using Discount Messages and Information Framing Messages

#### 3.2.1. Information Framing

Variations in the framing of information can exert tangible impacts on the intentions and decisions of target audiences [58]. Koop et al. [59] guided target audiences to process information through strategies including the provision of relevant knowledge, the offering of practical recommendations, the emphasis of social norms, and the elicitation of emotional responses, thereby intervening in the formation of their attitudes.

Pinon and Gambara categorized information frames into three types: the risk-based frame, the attribute-based frame, and the goal-based frame [60], and the present study employs the latter. The goal-based information frame is divided into two subtypes: the benefit frame and the loss frame. The benefit frame highlights the gains that stakeholders can acquire by taking action, whereas the loss frame underscores the losses that stakeholders may incur by failing to take action. The goal-based information frame, which integrates these two information presentation approaches, is frequently applied in the marketing communication of health-related products and services [61,62].

To date, it remains unclear whether the benefit frame or the loss frame is more effective at persuading consumers [63]. O’Keefe and Jensen [64] employed a meta-analytic approach to demonstrate that, for preventive behaviors, the gain frame and loss frame exert no statistically significant difference in their impacts on participants. Meyers-Levy and Maheswaran [65] examined the goal-based information frame in the context of healthy food and found that, compared to the gain frame, exposure to loss frame information made participants more sensitive to the prospect of forgoing a healthy diet if they opted against the target healthy food product. Therefore, the loss frame can better stimulate participants’ preference for the target healthy food. Accordingly, we propose:

**H6a.**  *Information framing messages for near-expired bread will have a significant positive impact on consumers’ purchase intentions*.

**H6b.**  *Information framing messages for near-expired bread will have a significant positive impact on consumers’ product attitudes*.

**H6c.**  *Information framing messages for near-expired bread will have a significant positive impact on consumers’ environmental external benefits*.

#### 3.2.2. Environmental Concern

Consumer environmental concern refers to individuals’ tendency to consider the environmental impacts of their consumption behaviors when purchasing goods or services, and it exerts a significant influence on their engagement in eco-friendly purchasing practices. Loebnitz and Grunert [66] found that environmentally conscious consumers are inclined to consider the implications of their actions for environmental protection and thus are more willing to adopt eco-friendly and sustainable consumption behaviors. Aschemann-Witzel et al. [67] further noted that consumers who purchase NEF tend to exhibit a dual focus on both environmental and economic considerations. When consumers’ personal views on environmental and economic issues align with their inherent preferences, the resultant impact on their behaviors will be more pronounced.

Although environmental concern may be an important factor influencing consumers’ purchase of NEF, some studies have indicated that it has a very limited impact on food waste [68]. In Study 2, to control for the effects of consumer environmental concern on purchase intentions, product attitudes, and environmental external benefits, this variable was treated as a covariate.

## 4. Methods

To empirically investigate the effects of different influencing factors on consumers’ purchase intentions toward near-expired bread, we first conducted Study 1. Subsequently, in Study 2, we further employed different levels of the price discount factor identified in Study 1 and adopted various types information framing messages to conduct an in-depth analysis of their impacts on consumers’ purchase intentions toward near-expired bread, their product attitudes, and environmental external benefits of purchasing such products.

Study 1 tested the consumers’ purchase intentions toward near-expired bread by using the SOR framework. The results revealed that price discounts and time remaining until expiration significantly influence consumers’ purchase intentions through perceived value and perceived risk. Normative beliefs with regard to food waste significantly influence consumers’ purchase intentions through moral satisfaction.

Study 2 adopted an information intervention approach to examine the effects of different price discounts and various types of information framing messages on consumers’ purchase intentions toward near-expired bread, their product attitudes, and their recognition of the environmental external benefits associated with purchasing near-expired bread. The results confirmed that discount messages significantly influence purchase intentions and product attitudes. Information framing messages significantly influence purchase intentions and environmental external benefits. Price discounts and information framing messages have a significant interactive effect on environmental external benefits.

### 4.1. Sample

Participants of study 1 were recruited from four major cities in China: Beijing, Shenzhen, Zhengzhou, and Chengdu. These four cities are representative of residents with distinct dietary habits and regional cultures across the country. A total of 1300 participants were recruited via both online platforms and paper-based questionnaires. After excluding 146 participants who failed the attention checks, the final valid sample consisted of 1154 participants. Among them, 913 responses were collected via paper-based questionnaires, and 241 via online ones. Online questionnaires were collected in collaboration with Power CX, a professional market research agency.

For Study 2, a total of 746 valid participants were recruited for the formal experiment, including 346 paper-based respondents and 400 online respondents. Participants were recruited from two major cities in China: Beijing and Zhengzhou. Of all valid respondents, 379 (50.8%) were recruited in Beijing, while 367 (49.2%) came from Zhengzhou.

When recruiting participants, we aimed to ensure a wide representation of respondents in terms of income, age, occupation, and other attributes. For this reason, we distributed paper questionnaires at various sites including bakeries, universities, banks, and hospitals, inviting participants to complete the questionnaires face-to-face on site to ensure broad sample coverage. The objective criteria used for participant recruitment were that they should be aged 18 or above, possess normal cognitive and reading abilities to complete the questionnaire independently, and voluntarily agree to take part in the study.

### 4.2. Materials and Measurement

Study 1 aims to examine how stimuli influence consumers’ purchase intentions toward NEF. Structural equation modeling (SEM) with Amos 26 was employed to analyze the hypothesized paths and test the relationships among the variables. The experimental design, and survey measures are elaborated upon in the subsequent sections.

The questionnaire consisted of a total of 31 items and measured the following constructs: sociodemographic characteristics (6 items), price discounts (5 items), time remaining until expiration (3 items), normative beliefs regarding food waste avoidance (3 items), moral satisfaction (3 items), perceived risk (3 items), perceived value (5 items), and purchase intentions (3 items).

Before finalizing Table 1 for the formal questionnaire, several items were removed from the scale. Specifically, two items were deleted from the price discount construct due to content redundancy with other items within the same dimension. One item was eliminated from the perceived risk construct for two reasons. First, its content overlapped with items measuring price discount. Second, according to face-to-face offline surveys with consumers, quality and safety concerns are their primary worries regarding near-expired bread; therefore, the perceived risk dimension in this study mainly focuses on quality risk and health risk. Participants rated their level of agreement with construct-related statements (Table 1) using a 7-point Likert scale, where higher scores indicated stronger agreement.

**Figure 2:**
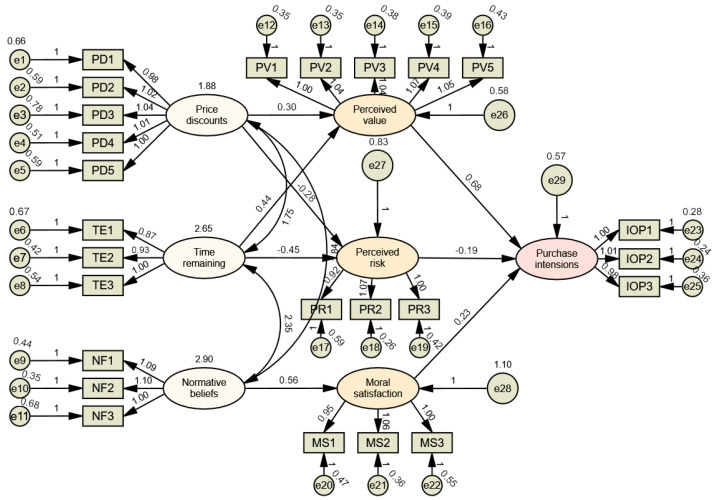
Research model.

This study identifies price discounts, time remaining until expiration, normative beliefs with regard to food waste avoidance, perceived value, perceived risk, and moral satisfaction as key factors influencing consumers’ purchase intentions toward near-expired bread. The research model is presented below (Figure 2).

**Figure 3:**
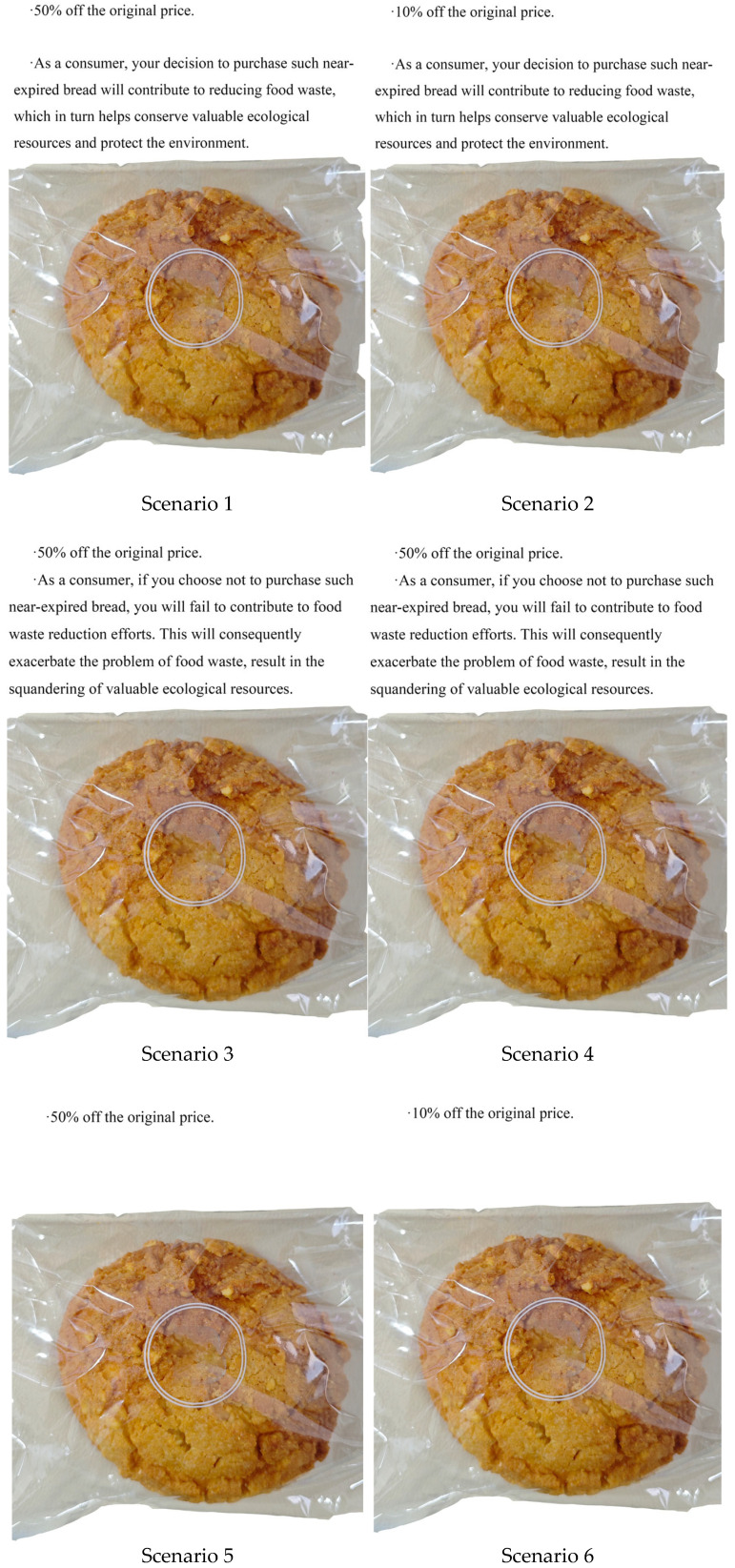
Each scenario consisted of two information components. The first paragraph corresponded to one of the two discount treatments (50% off or 10% off), and the second paragraph corresponded to one of the three information framing treatments (gain-framed, loss-framed, or no information treatment).

Study 2 adopted a 2 × 3 between-subjects factorial design, with the independent variables being discount level (high vs. low) and information framing type (gain-framed, loss-framed, or no message). Statistical analyses were performed using SPSS 27. A total of six experimental scenarios were generated (Figure 3). This scenario-based research design was employed to measure consumers’ purchase intentions, product attitudes, and environmental external benefits toward NEF. Details regarding the participants, experimental procedures, and survey measures are elaborated in the subsequent sections.

Manipulation checks are essential to verify that participants can distinguish between the message frames of different experimental scenarios before the formal experiment starts. A pretest was therefore conducted to perform these manipulation checks in the present study. The pretest was conducted using a paper-based questionnaire, and a total of 128 university students from Shenzhen were recruited as participants. Consistent with prior studies employing college students for manipulation checks [72], this study recruited university students as participants. They are easily accessible, help eliminate demographic confounding variables, exhibit higher acceptance of and attention to near-expired food [73], and ensure reliable manipulation checks. The inclusion criteria were as follows: aged 18 years or older, possessing normal cognitive and reading abilities, and providing voluntary informed consent to participate.

Questionnaires corresponding to four distinct scenarios were randomly distributed, such that each respondent was assigned to only one scenario questionnaire. Each scenario was allocated an equal sample size of 32 participants.

First, participants were instructed to read the scenario questionnaire carefully, then respond to manipulation check items, including: “Whether the price discount in the scenario would appeal to you?” and “If you purchased the NEF, would this exert a positive or negative impact on food waste reduction?”

The results of the manipulation checks revealed significant differences between scenarios with varying discount levels (M_50% off_ = 4.375; SD_50% off_ = 1.827; M_10% off_ = 3.125; SD_10% off_ = 1.431; F (1, 62) = 9.281; *p* = 0.003) and between different information framing conditions (M_gain-framed_ = 4.656; SD_gain-framed_ = 1.473; M_loss-framed_ = 3.125; SD_loss-framed_ = 1.362; F (1, 62) = 18.650; *p* < 0.001). These findings confirm that the manipulation was successful, because participants could effectively distinguish between the scenario designs corresponding to different levels of each independent variable.

The formal questionnaire assessed four key constructs: we used purchase intentions, product attitudes, and environmental external benefits as three dependent variables, and environmental concern as a covariate. Purchase intentions reflect consumers’ behavioral tendency to actually purchase near-expired bread, while product attitudes capture consumers’ affective and cognitive evaluations of the product. A positive attitude does not guarantee a high purchase intention. The dependent variable environmental external benefits reflects consumers’ recognition that purchasing near-expired bread can reduce food waste and protect the environment. Therefore, the two newly added dependent variables in Study 2, which are product attitudes and environmental external benefits, represent an extension and further in-depth investigation based on Study 1.

The questionnaire consisted of a total of 18 items and measured the following constructs: sociodemographic characteristics (6 items), purchase intentions (3 items), product attitudes (3 items), environmental external benefits (2 items), environmental concern (4 items).

Cronbach’s alpha coefficients for all latent variables exceeded 0.7 (Table 2), which indicates satisfactory internal consistency reliability.

Table 3 presents the detailed distribution of participants across each experimental group.

Participants were randomly assigned to a 2 (discount level: high vs. low) × 3 (information framing: gain-framed vs. loss-framed vs. no information) between-subjects factorial design, resulting in six experimental groups. First, participants were asked to read basic information that included the definition of NEF. Subsequently, they were randomly assigned to one of the six groups and provided with materials containing different message frames and an image of a near-expired bread (Figure 3). In the high-discount treatment group, participants received messages specifying a 50% discount, whereas the low-discount treatment group was presented with a 10% discount offer. The no-information treatment group served as the control condition, where participants received no additional message frames. In the gain-framed treatment, participants were informed that purchasing NEF would help reduce food waste and ultimately contribute to environmental protection. Conversely, in the loss-framed treatment, messages emphasized that failing to purchase NEF would exacerbate food waste and eventually lead to environmental degradation (Figure 3).

Discount treatments were determined based on preliminary on-site surveys conducted in four Chinese cities—Beijing, Tianjin, Jinan, and Zhengzhou—and these represent the commonly adopted discount levels in local bakeries, grocery stores, and supermarkets. During our on-site surveys in bakery shops and the bakery sections of large supermarkets in the above four cities, we visited approximately eight bakeries in each city. We observed that high discount rates generally stood at 50% off, while low discount rates were around 10% off. Accordingly, we set the high discount level at 50% and the low discount level at 10%.

The specific information provided was as follows:

Basic information

Near-expired bread is officially defined as bread that is close to its expiration date yet remains within the safe consumption window, and is thus safe for consumer consumption.

Please imagine that you are shopping for groceries in a store. You notice a display introducing near-expired bread on the bread shelf. Assume this near-expired bread has a total shelf life of 3 days, with only 1 day remaining until expiration. Please read the following information carefully and answer the questions based on your actual purchase preferences.

High discount

50% off the original price.

Low discount

10% off the original price.

Gain-framed information

As a consumer, your decision to purchase such near-expired bread will contribute to reducing food waste, which in turn helps conserve valuable ecological resources and protect the environment.

Loss-framed information

As a consumer, if you choose not to purchase such near-expired bread, you will fail to contribute to food waste reduction efforts. This will consequently exacerbate the problem of food waste and result in the squandering of valuable ecological resources.

Participants were asked to respond to questionnaire items pertaining to near-expired bread. Specifically, they rated their level of agreement with statement-based items measuring the constructs of interest (Table 2) using a 7-point Likert scale, where higher scores indicated stronger agreement. Study 2 was designed to assess participants’ purchase intentions, product attitudes, and environmental external benefits across the different experimental scenarios. In addition, environmental concern was incorporated as a covariate to control for its confounding effects on the dependent variables.

## 5. Results

### 5.1. Study 1

In order to validate the measurement model, three key steps need to be conducted: the assessment of item reliability, convergent validity, and discriminant validity [75]. The model comprises seven latent variables and 25 observed variables.

The composite reliability (CR) values of all latent variables exceed 0.7 (Table 4), indicating good internal consistency reliability. Cronbach’s alpha is another metric for assessing internal consistency; the alpha coefficients of all latent variables also surpass 0.7 (Table 1), which further confirms satisfactory internal consistency.

Convergent validity refers to the degree of positive correlation between a measure and other related measures of the same construct. It was assessed using the average variance extracted (AVE). A higher AVE value indicates a higher level of convergent validity, which implies that the indicators of a given construct are highly relevant and converge on that construct. As a general rule of thumb, an AVE value greater than 0.5 is considered acceptable. In this study, the AVE values for all latent variables exceeded 0.8 (Table 4), which means that over 80% of the variance in the observed indicators was explained by their respective constructs. This finding confirms that the measurement model meets the requirements for convergent validity [76].

Discriminant validity refers to the extent to which a construct (latent variable) is statistically distinguishable from other constructs, meaning that the items measuring distinct constructs should not exhibit high correlations with one another. This study assessed discriminant validity by comparing the square roots of the average variance extracted (AVE) values (presented in bold on the diagonal) with the latent variable correlation coefficients (reported in the off-diagonal cells). The results show that the square roots of the AVE values for all constructs exceed the corresponding latent variable correlations (Table 5). This finding indicates that items measuring different constructs are sufficiently uncorrelated, thereby confirming that the measurement model satisfies the criteria for discriminant validity [76].

Since Study 1 meets the criteria of item reliability, convergent validity, and discriminant validity, we proceed to examine the structural model, which estimates hypothesized paths with different latent constructs.

**Figure 4:**
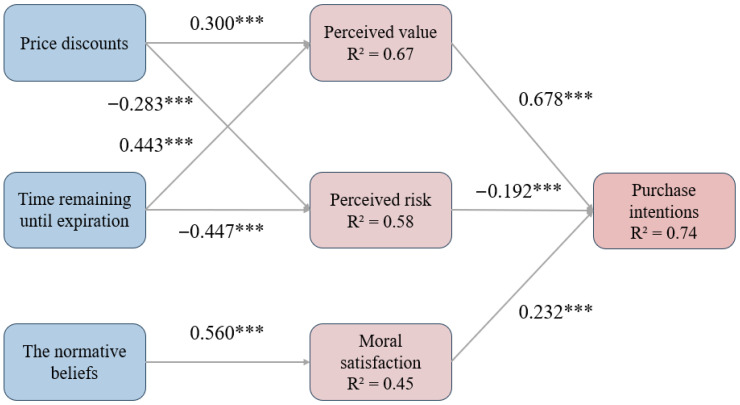
Measurement and structural model to predict the consumer’s purchase intentions. *** indicates a 1% significance level.

We use the SEM technique to calculate the values of the significance analysis of the structural model. Figure 4 presents a summary of the measurement and structural model. Table 6 shows the standard estimate of hypotheses and its stand errors and critical ratios with the associated *p*-value. From the results, we can support hypotheses H1a, H1b, H2a, H2b, H3a, H3b, H4a, and H5a. That is, there is a significant and positive association between price discounts and perceived value (Std. Estimate = 0.300, *p* < 0.001), a significant and negative association between price discounts and perceived risk (Std. Estimate = −0.283, *p* < 0.001), a significant and positive association between time left to expiration date and perceived value (Std. Estimate = 0.443, *p* < 0.001), a significant and negative association between time left to expiration date and perceived risk (Std. Estimate = −0.447, *p* < 0.001), a significant and positive association between the normative beliefs with regard to food waste avoidance and moral satisfaction (Std. Estimate = 0.560, *p* < 0.001), a significant and positive association between moral satisfaction and purchase intentions (Std. Estimate = 0.232, *p* < 0.001), a significant and positive association between perceived value and purchase intentions (Std. Estimate = 0.678, *p* < 0.001), and a significant and negative association between perceived risk and purchase intentions (Std. Estimate = −0.192, *p* < 0.001).

Study 1 also finds that price discounts and time left to expiration date increase consumers’ purchase intentions through increased perceived value and perceived risk. The normative beliefs with regard to food waste avoidance increase consumers’ purchase intentions through moral satisfaction. From the results, we can support hypotheses H3c, H4b, H4c, H5b, and H5c. That is, the normative beliefs with regard to food waste avoidance affect purchase intentions through moral satisfaction (path coeff. = 0.149, *p* = 0.000), price discounts affect purchase intentions through perceived value (path coeff. = 0.188, *p* = 0.000), time left to expiration date affect purchase intentions through perceived value (path coeff. = 0.329, *p* = 0.000), price discounts affect purchase intentions through perceived risk (path coeff. = 0.050, *p* = 0.001), and time left to expiration date affect purchase intentions through perceived risk (path coeff. = 0.094, *p* = 0.000). Table 7 shows the standard estimate of hypotheses related to mediating effect, and its path coefficient, lower bonds, and upper bonds with the associated *p*-value.

Both price discounts and the time remaining until expiration exert significant impacts on perceived value and perceived risk. This result reveals two key implications. First, while price discounts are a critical factor driving consumers to purchase near-expired bread, Chinese consumers still attach great importance to food safety when buying such products. This is because food safety in China goes far beyond mere technical deliberations and has evolved into a profound social and political issue embedded in the fabric of Chinese society [77]. Therefore, alongside price discounts, the remaining shelf life is equally important in their purchase decision-making.

Second, the near-expired bread market is still in the preliminary development stage in China, where there are no clear, unified mandatory standards or regulatory frameworks. As a result, consumers still harbor certain concerns regarding the quality and safety of near-expired bread when making purchase choices. Legal and regulatory reforms plus technology-driven solutions are critical to protecting consumer trust and the industry’s sustainable development [78].

Perceived value plays a crucial role in consumers’ acceptance of food close to its expiration date [79]. Grunert et al. [80] further demonstrated that the relationship between price and perceived value constitutes a key driver of consumers’ shopping choices. The significance of the mediating effect indicates that perceived value and perceived risk both play an important role in bridging consumers’ price discounts, time remaining until expiration, and their purchase intentions. Therefore, price discounts on near-expired bread and the assurance of its quality and safety can significantly affect consumers’ perceived value and perceived risk, and ultimately influence their purchase intentions.

The path through which consumers’ normative beliefs with regard to food waste avoidance positively influence their purchase intentions through moral satisfaction is significant for two possible reasons: first, the joint science popularization and publicity campaigns on anti-food waste launched by the government and media have significantly reshaped consumers’ subjective norms, leading them to perceive food waste as a social risk bearing on national food security and ecological sustainability [81]. Therefore, the recognition and acceptance of the concept of anti-food waste is an important reason for the significance of this path.

Second, to a certain extent, consumers could be aware of the connection between purchasing near-expired bread and reducing food waste. Therefore, after purchasing such bread, it aligns with their own normative beliefs with regard to food waste avoidance, thereby obtaining a certain degree of moral satisfaction and ultimately influencing their purchase intentions [27]. Food waste constitutes a critical issue at the societal level, particularly at the retail and consumer levels. Food waste avoidance is a value-based activity [82]. Therefore, compared to price discounts, shaping consumers’ perceptions of value regarding near-expired bread and promoting food waste avoidance are equally important.

### 5.2. Study 2

A two-way ANOVA was performed with discount and information framing as independent variables. Consumers’ purchase intentions, product attitudes, and environmental external benefits were treated as dependent variables. The results of ANOVA show that price discount treatments significantly impact purchase intentions (F (1, 740) = 68.087, *p* < 0.001), thus supporting H1c. Price discount treatments significantly impact product attitudes (F (1, 740) = 9.854, *p* = 0.002), thus supporting H1d. Information framing treatments significantly impact purchase intentions (F (2, 740) = 10.833, *p* < 0.001). Thus, H6a was supported. Information framing treatments significantly impact product attitudes (F (2, 740) = 3.430, *p* = 0.033), thus supporting H6b. Information framing treatments significantly impact environmental external benefits (F (2, 740) = 77.314, *p* < 0.001). Thus, H6c was supported.

The results reveal a two-way interaction on environmental external benefits. The interaction between discounts and information framing was significant (F (2, 740) = 7.488, *p* < 0.001). On the contrary, the results do not reveal a two-way interaction on purchase intentions. The interaction between discounts and information framing was not significant (F (2, 740) = 0.518, *p* = 0.596). The results do not reveal a two-way interaction on product attitudes. The interaction between discounts and information framing was not significant (F (2, 740) = 1.272, *p* = 0.281). The results of ANOVA are shown in Table 8.

Based on the results of the simple effect test, when the discount rate is under 50%, gain-framed messages do not raise significantly higher environmental external benefits than loss-framed messages (mean difference = 0.034, *p* = 0.872). Gain-framed messages raise significantly higher environmental external benefits than no message (mean difference = 2.166, *p* < 0.001). Loss-framed messages raise significantly higher environmental external benefits than no message (mean difference = 2.132, *p* < 0.001). When the discount rate is under 10%, gain-framed messages did not raise significantly lower environmental external benefits than loss-framed messages (mean difference = −0.001, *p* = 0.997). Gain-framed messages raise significantly higher environmental external benefits than no message (mean difference = 1.129, *p* < 0.001). Loss-framed messages raise significantly higher environmental external benefits than no message (mean difference = 1.129, *p* < 0.001). The results of the simple effect test of environmental external benefits are shown in Table 9.

The post hoc test (Table 10) showed that gain-framed messages did not raise significantly lower purchase intentions than loss-framed messages (mean difference = −0.209, *p* = 0.165). Gain-framed messages raise significantly higher purchase intentions than no message (mean difference = 0.464, *p* = 0.003). Loss-framed messages raise significantly higher purchase intentions than no message (mean difference = 0.673, *p* < 0.001). Gain-framed messages did not raise significantly higher product attitudes than loss-framed messages (mean difference = −0.240, *p* = 0.190). Gain-framed messages did not raise significantly higher product attitude than no message (mean difference = 0.205, *p* = 0.201). Loss-framed messages raise significantly higher purchase intentions than no message (mean difference = 0.408, *p* = 0.011). Gain-framed messages did not raise significantly higher environmental external benefits than loss-framed messages (mean difference = 0.021, *p* = 0.886). Gain-framed messages raise significantly higher environmental external benefits than no message (mean difference = 1.651, *p* < 0.001). Loss-framed messages raise significantly higher environmental external benefits than no message (mean difference = 1.630, *p* < 0.001).

ANCOVA was performed with environmental concern as a covariate. The results of ANCOVA show that environmental concern significantly impacts purchase intentions (F (0, 740) = 97.970, *p* < 0.001). Environmental concern significantly impacts product attitudes (F (0, 740) = 131.420, *p* < 0.001) and environmental external benefits (F (0, 340) = 188.012, *p* < 0.001). Price discount treatments significantly impact purchase intentions (F (1, 740) = 72.542, *p* < 0.001), thus supporting H1c. Price discount treatments significantly impact product attitudes (F (1, 740) = 9.657, *p* = 0.002), thus supporting H1d. Information framing treatments significantly impact purchase intentions (F (2, 740) = 8.460, *p* < 0.001), thus supporting H6a. Information framing treatments did not significantly impact product attitude (F (2, 740) = 2.190, *p* = 0.113); thus, H6b was not supported. Information framing treatments significantly impact environmental external benefits (F (2, 740) = 79.012, *p* < 0.001); thus, H6c was supported.

The results reveal a two-way interaction between environmental external benefits. The interaction between discounts and information framing was significant (F (2, 740) = 9.696, *p* < 0.001) when the main effect was significant. The results of ANCOVA are shown in Table 11. On the contrary, the results do not reveal a two-way interaction with purchase intention; thus, the interaction between discounts and information framing was not significant (F (2, 740) = 0.637, *p* = 0.529). The results also do not reveal a two-way interaction with product attitudes; thus, the interaction effect between discounts and information framing was not significant (F (2, 740) = 1.643, *p* = 0.194).

Merely relying on social trust is not sufficient to boost consumers’ purchase intention or behavior toward food with cosmetic defects. However, consumers with strong concerns about environmental issues and high levels of social trust are more inclined to purchase such food. Increasing consumers’ awareness of environmental issues—especially among those with low social trust—may incentivize more people to buy food with cosmetic defects [66]. Furthermore, the ANCOVA results indicate that Chinese consumers consider not only price but also environmental impacts when purchasing near-expired bread.

China has achieved remarkable economic development over the past several decades, but the ecological and environmental costs incurred in the process cannot be ignored. For instance, microplastic pollution in China’s agricultural soils is severe [83]. Therefore, reducing food waste and improving the ecological environment are critical and pressing. Meanwhile, as China’s economy continues to grow, Chinese consumers have seen a marked increase in their awareness of environmental protection and food waste reduction. A growing number of Chinese consumers are gradually recognizing that environmental protection and food waste reduction are not merely the responsibility of the government, but a commitment that every individual can contribute to through behavioral changes. Wu et al. [84] found that compared to Tokyo and Bangkok, the amount of food leftovers generated by diners in Beijing, Shanghai, and Wuhan is significantly higher. This suggests that there is still considerable room for improvement in the awareness of food waste reduction and environmental protection among Chinese consumers as a whole.

The non-significant interaction between discount messages and information-framing messages on purchase intentions and product attitudes in both ANOVA and ANCOVA indicates that, for consumers, discounts activate an “economic utility” route (price–quality trade-off), whereas information-framing activates a “moral or environmental” route (self-identity). The two routes are driven by distinct motivations, making synergy unlikely.

The results of post hoc tests indicate that, regarding product attitudes, loss-framed messages are more effective at enhancing consumers’ awareness of the urgency and link between purchasing near-expired bread and reducing food waste and protecting the environment compared to gain-framed messages, thereby positively enhancing their product attitudes towards near-expired bread. Therefore, in the future, when retailers sell NEF, they can attach greater importance to the use of loss-framed messages centered on environmental protection and food waste reduction to more effectively boost the sales of such food.

## 6. Conclusions

### 6.1. General Discussion

This study demonstrates that price discounts and remaining shelf-life significantly influence consumers’ purchase intentions toward near-expired bread through two distinct psychological pathways: enhanced perceived value and reduced perceived risk. Additionally, normative beliefs regarding food waste avoidance positively affect purchase intentions by increasing consumers’ moral satisfaction.

Our findings further reveal that gain-framed versus loss-framed information messages exert significant effects on consumers’ purchase intentions, product attitudes, and perceptions of environmental external benefits. Similarly, price discount messages at different levels (50% vs. 10% off) significantly shape purchase intentions and product attitudes, though they do not influence perceived environmental external benefits. The covariate, environmental concern, consistently predicts all three outcome variables: purchase intentions, product attitudes, and environmental external benefits. Notably, we identify an interactive effect between price discount messages and information framing on consumers’ perceptions of environmental external benefits, suggesting that the combination of pricing and messaging strategies matters beyond their individual effects.

Consistent with existing literature [9] on price discounts, this study further reveals that even when the discount reaches 50% off, consumers still show positive purchase intentions toward near-expired bread. Such a large discount does not lead consumers to doubt the product quality. The findings of this research indicate that the time remaining until expiration of near-expired bread also significantly influences consumers’ purchase intentions, which is consistent with previous literature [10]. Regarding environmental concern, the results show that consumers who are more aware of environmental protection and food waste reduction exhibit stronger purchase intentions toward near-expired bread driven by such altruistic considerations, aligning with prior literature [11,20,27]. Our findings indicate that for Chinese consumers, while price is a key factor influencing their purchase of near-expired bread, concerns about the quality and safety of such bread, the motivation to reduce food waste, and considerations for environmental impact also serve as important determinants of their purchasing decisions.

### 6.2. Practical Implications

The findings of this study also offer practical implications for both retailers and policymakers aiming to promote regular consumption of near-expired food and advocate green consumption practices.

For policymakers, first, the criteria and detailed rules for identifying near-expired food should be formulated in accordance with local conditions such as climatic factors across different regions. Meanwhile, classification thresholds for near-expired products should be established hierarchically based on inherent food characteristics, including product category, storage conditions, and shelf-life duration. Second, the management and sales of near-expired food should be aligned with the Anti-Food Waste Law of the People’s Republic of China and incorporated into its legal framework, so as to guarantee the safety of near-expired food and ensure unobstructed and standardized sales channels.

For retailers, first, they should prominently emphasize the reliable quality and safety of NEF products in their marketing campaigns. By adopting rigorous product management protocols, retailers can substantially improve the quality and safety of NEF offerings, thereby reducing consumers’ reluctance to purchase such products due to quality and safety concerns. Second, retailers could highlight the positive contribution of purchasing NEF products to food waste reduction. Such value-oriented messaging helps enhance consumers’ purchase intentions toward NEF items [85].

### 6.3. Limitations and Future Research Directions

This study has several limitations that point to promising directions for future inquiry. First, this study selected consumers from four representative cities in China and it did not cover economically underdeveloped regions, especially the vast number of rural consumers in China. In addition, due to time and cost constraints, the manipulation check associated with Study 2 was conducted using students from a single university in Shenzhen. College students tend to be more price-sensitive. Therefore, the findings of the manipulation check should be generalized cautiously to older and less educated consumer groups. Future research is suggested to incorporate a more diverse sample to conduct a more comprehensive investigation. Second, while our investigation focused on the purchase stage of near-expired bread, a complete understanding of food waste reduction requires examining post-purchase behaviors. Future research should explore how consumers manage and potentially waste these products after bringing them home, thereby completing the logical chain from acquisition to disposal. Third, although the information intervention in our study examined price discounts and environmental factors that consumers care about, it did not investigate quality and safety perceptions regarding near-expired bread. Future research may conduct information intervention experiments using messages related to best-before dates and quality and safety, to examine how such information influences consumers’ purchase intention toward near-expired bread and other related factors. Fourth, although this study focuses on consumers’ purchasing behavior toward near-expired bread, it does not investigate retailers, another key participant in the consumption process. Future research can explore the operation models of near-expired food from the retailer’s perspective. Addressing these limitations in future work would not only strengthen the evidence base but also contribute to a more comprehensive understanding of consumer behavior throughout the near-expired food lifecycle.

## Figures and Tables

**Table 1:** Scales and items used as predictors for study 1.

Scales and Items		Source
Price discounts (α = 0.939, M = 4.756, SD = 1.605)		
1	I frequently buy food close to the best-before date, if it is offered at a lower price.	Aschemann-Witzel [33]
2	I look for ads in the newspaper for store specials or purchase food that is on discount.	Aschemann-Witzel [33]
3	I would rather wait buying groceries, until they are on sale	Aschemann-Witzel [67]
4	At the supermarket, I look for items which are on sale.	Aschemann-Witzel [67]
5	I am prone to buying groceries on sale.	Aschemann-Witzel [67]
Time remaining until expiration (α = 0.930, M = 5.060, SD = 1.698)		
1	Whether I buy near-expired bread depends on its time left to expiration date.	Developed for this study
2	I prefer to buy near-expired bread that is close to its expiration date but still meets my requirements for the time left to expiration date.	Developed for this study
3	I prefer to buy near-expired bread that is close to its expiration date but has more days left until it expires.	Developed for this study
Normative beliefs with regard to food waste avoidance (α = 0.953, M = 5.285, SD = 1.944)		
1	I always try to eat all purchased foods.	V.H. Visschers et al. [69]
2	I try to waste no food at all.	V.H. Visschers et al. [69]
3	I always eat what is on my plate.	Aschemann-Witzel [33]
Moral satisfaction (α = 0.929, M = 4656, SD = 1.583)		
1	Buying near-expired bread makes me feel like a better person.	Zhang et al. [27]
2	Buying near-expired bread feels like a personal contribution to a good cause.	Zhang et al. [27]
3	Buying near-expired bread is in line with my moral standards.	Developed for this study
Perceived risk (α = 0.915, N = 3, M = 5.078, SD = 1.446)		
1	The nutrition or functionality of near-expired bread is lower compared to that of non-near-expired bread.	Cao [70]
2	Eating near-expired bread increases the risk of getting sick.	Cao [70]
3	Eating near-expired bread can be harmful to health.	Cao [70]
Perceived value (α = 0.961, M = 4.910, SD = 1.503)		
1	Near-expired bread and non-near-expired bread look identical in appearance.	Aschemann-Witzel [33]
2	Near-expired bread and non-near-expired bread taste the same.	Aschemann-Witzel [33]
3	Consuming near-expired bread and non-near-expired bread has the same impact on human health.	Aschemann-Witzel [33]
4	The price-value ratio of near-expired bread is fair.	Aschemann-Witzel [33]
5	All things considered, the quality of near-expired bread is the same as that of non-near-expired bread.	Aschemann-Witzel [33]
Purchase intentions (α = 0.960, M = 4.971, SD = 1.631)		
1	I am very likely to purchase near-expired bread.	Liu et al. [71]
2	I am willing to purchase near-expired bread.	Liu et al. [71]
3	I will purchase near-expired bread if it is available to me.	Developed for this study

**Table 2:** Scales and items used as predictors for study 2.

Scales and Items		Source
Purchase intentions (α = 0.958, M = 4.019, SD = 1.869)		
1	I have a high probability of buying near-expired bread.	Liu et al. [71]
2	I have willingness to purchase near-expired bread.	Liu et al. [71]
3	If I had access to purchase near-expired bread, I would purchase it.	Developed for this study
Product attitudes (α = 0.952, M = 4.069, SD = 1.863)		
1	My attitude towards this near-expired bread is positive.	Biasini et al. [74]
2	My attitude towards this near-expired bread is favorable.	Biasini et al. [74]
3	My attitude towards this near-expired bread is that I like it.	Biasini et al. [74]
Environmental external benefits (α = 0.954, M = 4.468, SD = 1.883)		
1	I believe buying this near-expired bread contributes to environmental sustainability.	Developed for this study
2	Purchasing this near-expired bread helps promote environmental protection.	Developed for this study
Environmental concern (α = 0.865, M = 4.992, SD = 1.548)		
1	I typically take the impact of food waste on the ecological environment into account.	Aschemann-Witzel [67]
2	I am environmentally responsible.	Aschemann-Witzel [67]
3	Even if purchasing environmentally friendly food (e.g., near-expired products) causes inconvenience in my daily life, I am prepared to tolerate it for environmental protection.	Aschemann-Witzel [67]
4	When making food purchases, I tend to incorporate environmental impacts into my decision-making.	Aschemann-Witzel [67]

**Table 3:** The description of stimulus and its sample size for study 2.

Scenario Number	Discounts	Information Framing	Sample Size
1	50% off	Gain-framed	126
2	50% off	Loss-framed	125
3	50% off	No message	119
4	10% off	Gain-framed	130
5	10% off	Loss-framed	134
6	10% off	No message	112

**Table 4:** Reliability measurements.

	Items	Standard Loading	Composite Reliability	AVE
Price discounts (PD)	PD1	0.829	0.939	0.756
	PD2	0.875		
	PD3	0.856		
	PD4	0.909		
	PD5	0.876		
Time remaining until expiration (TE)	TE1	0.867	0.931	0.819
	TE2	0.942		
	TE3	0.905		
Normative beliefs with regard to food waste avoidance (NF)	NF1	0.945	0.953	0.871
	NF2	0.949		
	NF3	0.905		
Perceived value (PV)	PV1	0.914	0.961	0.833
	PV2	0.924		
	PV3	0.914		
	PV4	0.906		
	PV5	0.905		
Perceived risk (PR)	PR1	0.854	0.931	0.818
	PR2	0.953		
	PR3	0.903		
Moral satisfaction (MS)	MS1	0.895	0.929	0.815
	MS2	0.942		
	MS3	0.869		
purchase intentions (IOP)	IOP1	0.944	0.960	0.889
	IOP2	0.956		
	IOP3	0.928		

**Table 5:** Discriminant validity assessment.

	IOP	MS	PR	PV	NF	TE	PD
IOP	**0.943**						
MS	0.688	**0.903**					
PR	−0.732	−0.587	**0.904**				
PV	0.844	0.678	−0.784	**0.913**			
NF	0.815	0.654	−0.732	0.794	**0.933**		
TE	0.755	0.603	−0.702	0.754	0.819	**0.905**	
PD	0.754	0.618	−0.668	0.721	0.773	0.783	**0.869**

Diagonals in bold indicate the square root of each constructs’ AVE. Off-diagonals are the latent variable correlations.

**Table 6:** Significance analysis of the structural model.

Hypotheses	Path	Standard Estimate	S.E.	C.R.	Hypothesis
H1a	PD → PV	0.300 ***	0.033	9.003	Supported
H1b	PD → PR	−0.283 ***	0.040	−7.103	Supported
H2a	TE → PV	0.443 ***	0.029	15.412	Supported
H2b	TE → PR	−0.447 ***	0.034	−13.112	Supported
H3a	NF → MS	0.560 ***	0.023	24.537	Supported
H3b	MS → IOP	0.232 ***	0.022	10.653	Supported
H4a	PV → IOP	0.678 ***	0.028	23.910	Supported
H5a	PR → IOP	−0.192 ***	0.025	−7.766	Supported

*** indicates a 1% significance level.

**Table 7:** Mediation effect analysis results.

Hypothesis	Path	Path Coefficient	Lower Bonds	Upper Bonds	*p*-Value
H3c	NF → MS → IOP	0.149	0.102	0.199	0.000
H4b	PD → PV → IOP	0.188	0.102	0.271	0.000
H4c	TE → PV → IOP	0.329	0.241	0.426	0.000
H5b	PD → PR → IOP	0.050	0.019	0.091	0.001
H5c	TE → PR → IOP	0.094	0.053	0.147	0.000

**Table 8:** Results of ANOVA.

Independent Variables	Dependent Variables	Mean Square	F Value	*p*-Value
Price discount messages (PD)	Purchase intentions	197.405	68.087	<0.001
	Product attitudes	30.573	9.854	0.002
	Environmental external benefits	5.360	1.937	0.164
Information framing messages (IF)	Purchase intentions	31.407	10.833	<0.001
	Product attitudes	10.641	3.430	0.033
	Environmental external benefits	213.991	77.314	<0.001
PD × IF	Purchase intentions	1.502	0.518	0.596
	Product attitudes	3.947	1.272	0.281
	Environmental external benefits	20.725	7.488	<0.001

**Table 9:** A simple effect test of environmental external benefits.

Dependent Variables	Factors	IF Type 1	IF Type 2	Mean Difference	Standard Error	*p*-Value
Environmental external benefits	50% off	Gain-framed	Loss-framed	0.034	0.210	0.872
		Gain-framed	No message	2.166	0.213	<0.001
		Loss-framed	No message	2.132	0.213	<0.001
	10% off	Gain-framed	Loss-framed	−0.001	0.205	0.997
		Gain-framed	No message	1.129	0.214	<0.001
		Loss-framed	No message	1.129	0.213	<0.001

**Table 10:** Post hoc test.

Dependent Variables	IF Type 1	IF Type 2	Mean Difference	*p*-Value	95% Confidence Interval	
					Lower Bond	Upper Bond
Purchase intentions	Gain-framed	Loss-framed	−0.209	0.165	−0.503	0.086
	Gain-framed	No message	0.464	0.003	0.161	0.767
	Loss-framed	No message	0.673	<0.001	0.370	0.975
Product attitudes	Gain-framed	Loss-framed	−0.204	0.190	−0.508	0.101
	Gain-framed	No message	0.205	0.201	−0.109	0.518
	Loss-framed	No message	0.408	0.011	0.095	0.721
Environmental external benefits	Gain-framed	Loss-framed	0.021	0.886	−0.267	0.309
	Gain-framed	No message	1.651	<0.001	1.354	1.947
	Loss-framed	No message	1.630	<0.001	1.334	1.925

**Table 11:** Results of ANCOVA.

Independent Variables	Dependent Variables	Mean Square	F Value	*p*-Value
Environmental concern	Purchase intentions	251.136	97.970	<0.001
	Product attitudes	346.643	131.420	<0.001
	Environmental external benefits	415.402	188.012	<0.001
Price discount messages (PD)	Purchase intentions	185.954	72.542	<0.001
	Product attitudes	25.473	9.657	0.002
	Environmental external benefits	3.199	1.448	0.229
Information framing messages (IF)	Purchase intentions	21.688	8.460	<0.001
	Product attitudes	5.775	2.190	0.113
	Environmental external benefits	174.572	79.012	<0.001
PD × IF	Purchase intentions	1.633	0.637	0.529
	Product attitudes	4.333	1.643	0.194
	Environmental external benefits	21.424	9.696	<0.001

## Data Availability

The original contributions presented in the study are included in the article, further inquiries can be directed to the corresponding author.
